# Novel identification and characterisation of Transient receptor potential melastatin 3 ion channels on Natural Killer cells and B lymphocytes: effects on cell signalling in Chronic fatigue syndrome/Myalgic encephalomyelitis patients

**DOI:** 10.1186/s40659-016-0087-2

**Published:** 2016-05-31

**Authors:** T. Nguyen, D. Staines, B. Nilius, P. Smith, S. Marshall-Gradisnik

**Affiliations:** The National Centre for Neuroimmunology and Emerging Diseases, Menzies Health Institute, Griffith University, Parklands Drive, Southport, Mailbox 68, Gold Coast, 4222 Australia; School of Medical Science, Griffith University, Gold Coast, Australia; Department of Molecular Cell Biology, Laboratory of Ion Channel Research, KU Leuven University, 49 Herestraat, Leuven, B-3000 Belgium

**Keywords:** Chronic fatigue syndrome, Transient receptor potential, Calcium signalling, Myalgic encephalomyelitis

## Abstract

**Background:**

Transient receptor potential melastatin 3 (TRPM3) cation channels are ubiquitously expressed by multiple cells and have an important regulatory role in calcium-dependent cell signalling to help maintain cellular homeostasis. TRPM3 protein expression has yet to be determined on Natural Killer (NK) cells and B lymphocytes. Multiple single nucleotide polymorphisms have been reported in *TRPM3* genes from isolated peripheral blood mononuclear cells, NK and B cells in Chronic fatigue syndrome/Myalgic encephalomyelitis (CFS/ME) patients and have been proposed to correlate with illness presentation. The object of the study was to assess TRPM3 surface expression on NK and B lymphocytes from healthy controls, followed by a comparative investigation examining TRPM3 surface expression, and cytoplasmic and mitochondrial calcium influx in CD19^+^ B cells, CD56^bright^ and CD56^dim^ cell populations from CFS/ME patients.

**Results:**

TRPM3 cell surface expression was identified for NK and B lymphocytes in healthy controls (CD56^bright^ TRPM3 35.72 % ± 7.37; CD56^dim^ 5.74 % ± 2.00; B lymphocytes 2.05 % ± 0.19, respectively). There was a significant reduction of TRPM3 surface expression on CD19^+^ B cells (1.56 ± 0.191) and CD56^bright^ NK cells (17.37 % ± 5.34) in CFS/ME compared with healthy controls. Anti-CD21 and anti-IgM conjugated biotin was cross-linked with streptavidin,and subsequently treatment with thapsigargin. This showed a significant reduction in cytoplasmic calcium ion concentration in CD19^+^ B lymphocytes. CD56^bright^ NK cells also had a significant decrease in cytoplasmic calcium in the presence of 2-APB and thapsigargin in CFS/ME patients.

**Conclusions:**

The results from this preliminary investigation identify, for the first time, TRPM3 surface expression on both NK and B lymphocytes in healthy controls. We also report for the first time, significant reduction in TRPM3 cell surface expression in NK and B lymphocytes, as well as decreased intracellular calcium within specific conditions in CFS/ME patients. This warrants further examination of these pathways to elucidate whether TRPM3 and impaired calcium mobilisation has a role in CFS/ME.

**Electronic supplementary material:**

The online version of this article (doi:10.1186/s40659-016-0087-2) contains supplementary material, which is available to authorized users.

## Background

Mammalian Transient receptor potential ion channels (TRPs) are comprised of six main groups including the TRPA (ankyrin), TRPC (canonical), TRPM (melastatin), TRPML (mucolipin), TRPP (polycystin) and TRPV (vanilloid) [1]. These have been found to have different biological functions, activation mechanisms and differential expression on tissues throughout the body [2]. TRPs are extensively expressed on almost all cells and their dysregulation has been associated with pathological conditions and diseases such as cancer, glomerulosclerosis, pain syndrome, Olmsted syndrome, mucolipidoses, and polycystic kidney disease [3–5].

TRPs are activated following fluctuations or deviations in the cellular environment, including pathogens, temperature, pressure, chemicals, oxidation and reduction, toxins, osmolarity, and pH [6, 7]. TRPs are calcium (Ca^2+^) permeable cation channels that act as an excitatory signal when open to induce depolarisation of the cell and allow Ca^2+^ influx. Ca^2+^ plays a role in intracellular signalling pathways, contributing to a number of cellular processes, for example cell division, apoptosis and transcriptional events [8]. TRPM3 protein expression has been identified on pancreatic β cells, [9] as well as on cells in the central nervous system and the eye [2]. Chronic fatigue syndrome, also referred to as Myalgic encephalomyelitis (CFS/ME) (is a disorder marked by unexplained, debilitating fatigue accompanied by a range of symptoms relating to multiple physiologies [12]. CFS/ME patients may have reactions to a number of environmental and biological factors [13]. TRP channels may be targeted during inflammatory reactions as they are easily activated in the presence of irritants, inflammatory products, and xenobiotic toxins. Symptoms relating to sensitivity to toxins and irritants have also been associated with CFS/ME [14], however the causes of these sensitivities have not been fully investigated.

Immunological dysfunction is commonly reported in CFS/ME. A significant reduction in NK cytotoxic activity is a consistent finding in CFS/ME patients compared with healthy controls [15–26]. Furthermore, a significant increase in CD20^+^CD5^+^ B cells and CD19^+^IgM^+^ B cells has been reported in CFS/ME patients compare with healthy controls [27–31]. Atypical SNPs of *TRP* genes, particularly SNPs in *TRPM3* from peripheral blood mononuclear cells, NK and B cells have been recently reported in CFS/ME groups compared with healthy controls [10, 11, 32]. Thus, the first aim of this preliminary investigation was to identify TRPM3 surface protein expression on NK and B lymphocytes from healthy controls, using flow cytometric protocols. To date, there has been no previous evidence of this expression. Furthermore, protocols used to identify TRP receptors on specific cell subsets are based on spectroscopy and crystallography methods. The second aim of this study was to conduct a comparative assessment between CFS/ME and healthy controls for TRPM3 surface protein expression and changes in intracellular calcium influx in NK and B lymphocytes. Perturbations of TRPM3 expression and consequent changes in intracellular calcium influx could provide further knowledge as to the possibility of TRPM3 contributing to the pathogenesis of CFS/ME.

## Methods

### Subjects

This study consisted of 17 CFS/ME patients that were aged matched with 19 healthy controls (Table 1). Of the 17 CFS/ME patients (age = 48.68 ± 1.06 years), 14 (82 %) were females and 3 (18 %) were males. The 19 healthy controls (age = 46.48 ± 1.22 years) comprised 13 (68 %) females and 6 (32 %) males. CFS/ME patients were defined according to the Fukuda criteria, which required persistent, ongoing fatigue for at least 6 months accompanied by at least 4 of the following: post-exertional malaise, impaired short term memory or concentration, unrefreshed sleep, headaches, sore throat, tender lymph nodes, muscle pain, and joint pain [12]. The average duration of illness of this group was 8.4 years and reported no alternative disease or illness that would explain the onset of their symptoms. Any patients on potential immune modulating medication were excluded from this study. Healthy controls did not meet any CFS/ME criteria. All participants in this study were of Caucasian decent and residents of Australia at the time of blood collection. Exclusion criteria for all participants involved in this study were those who were smokers, pregnant or breastfeeding.

80 ml of whole blood was collected from all participants into EDTA blood tubes. Written consent was obtained from all participants prior to sample collection. Pathology testing parameters (full blood count, electrolytes, high sensitive C reactive protein and erythrocyte sedimentation rate) were performed on all participants. No significant differences in these pathology testing parameters were identified between groups (data not shown). This investigation was conducted under the Griffith University Ethics reference number (HREC/15/QGC/63).

### Peripheral blood mononuclear cells preparations

Peripheral blood mononuclear cells (PBMCs) were isolated from 40 ml EDTA blood after obtaining participants’ written consent. PBMCs were isolated using Ficoll density gradient centrifugation (Life technologies) as previously described [22]. PBMCs were counted using Trypan blue and concentration was adjusted to 1 × 10^7^ cells in 100 μl in Dulbecco’s phosphate-buffered saline (DPBS) with Ca^2+^ and magnesium (Life technologies) for all assays.

### TRPM3 immunophenotyping assay

Due to the constitutive active property of TRPM3 protein and readily available anti-TRPM3 antibody, this study utilised anti-TRPM3 antibody to measure protein expression as the antibody binds to the extracellular surface of the protein structure. Initially, PBMCs were incubated in 20 μl of FCR blocking reagent (Miltenyi Biotec) for 10 min at room temperature and washed with phosphate buffer saline (PBS) prior to centrifuge at 400*g* for 5 min. Supernatant was removed and incubated with primary fluorochrome labelled antibodies (CD19-BV421, CD3-PerCP, CD56-BV421 and CD16-APC Cy7, purchase from BD Bioscience) for 30 min at room temperature in the dark. Labelled cells were washed and incubated with 10 μg final concentration of goat anti-human TRPM3 antibody for 30 min. This was followed by a wash and resuspension in a final concentration of 5 % (v/v) of Bovine Serum Albumin (Sigma) for 30 min. Cells were washed again and incubated with 5 μg final concentration of donkey anti-goat IgG FITC (Santa Scruz) for 30 min. Cells were washed and resuspended in 200 μl of staining buffer (BD Bioscience) and acquired at 50,000 events using LSRFortessa X-20 (BD Bioscience).

### LSR Fortessa X-20 flow cytometry analysis

**Fig. 1:**
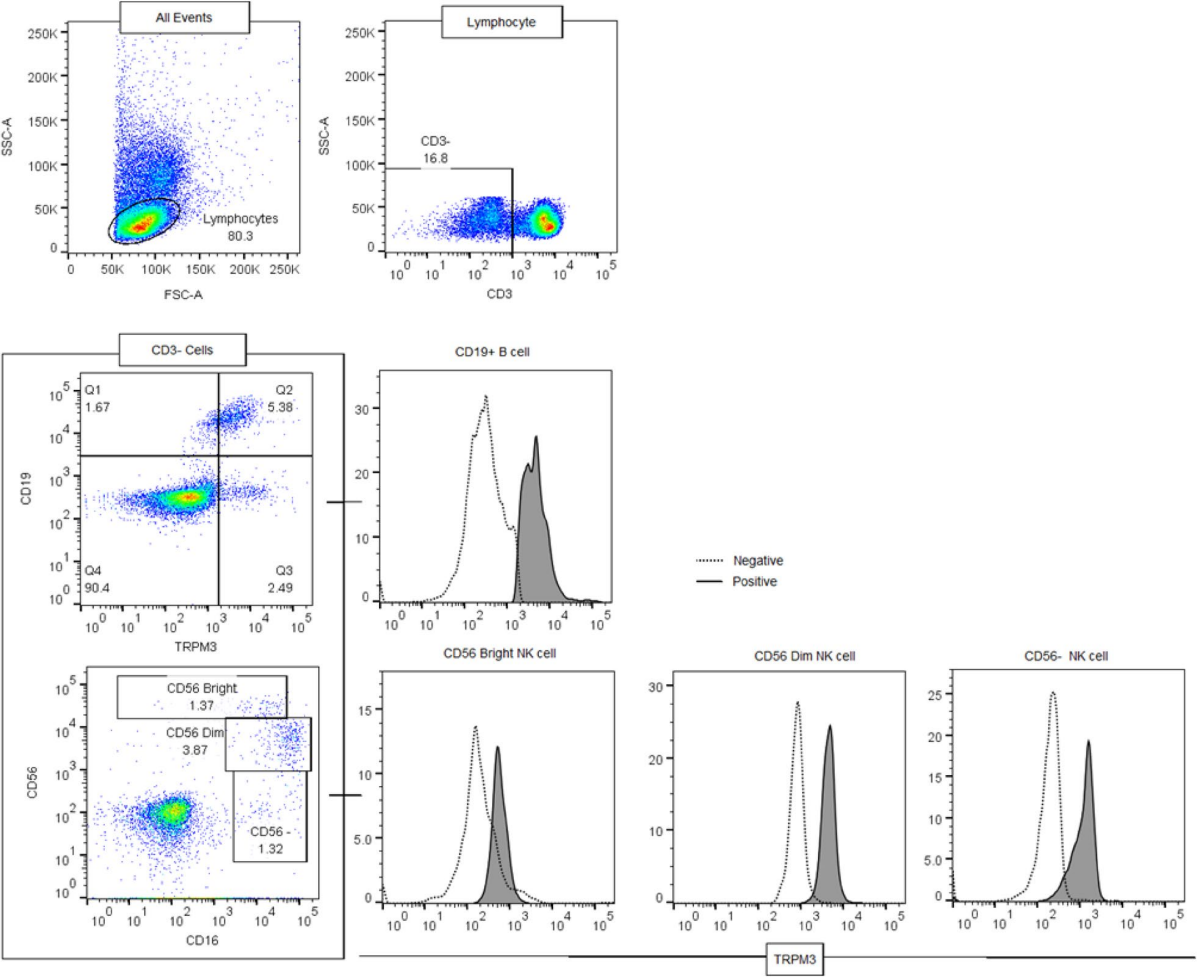
Immunophenotype of TRPM3 channel surface expression on B and NK cells by flow cytometry. Total B cells were identified using Anti-CD19. NK cells were characterised using the expression of CD56^Bright^CD16^Dim/−^ NK cells, CD56^Dim^CD16^Bright/+^ NK cells and CD56^−^CD16^+^ NK cells, followed by, identification of TRPM3 surface expression

Lymphocyte populations were identified using forward scatter and side scatter (FSC, SSC) dot plots. Exclusions were CD3^+^ cells and only CD3^−^ lymphocytes were further used to characterised B lymphocytes and NK cell subset populations using CD19, CD56 and CD16 as previously described [22, 33]. Total B cells were identified as CD19^+^, whereas NK cell subsets were characterised using the expression of CD56^Bright^CD16^Dim/−^ NK cells, CD56^Dim^CD16^Bright/+^ NK cells and CD56^−^CD16^+^ NK cells (Fig. 1). TRPM3 expression was measured as percentage of parent cells (%) and mean fluorescence intensity (MFI) (Additional file 1: Table S1). LSRFotessa X-20 flow cytometry was utilised for sequential determination of cytoplasmic calcium [Ca^2+^]_C_ and mitochondrial [Ca^2+^]_M_, to help compare cytoplasmic or mitochondrial Ca^2+^ influx kinetics in B lymphocytes and NK cells. Characterising kinetic measurements using median florescence of Fura-AM or Rhod-2 AM dye were used and smoothing curve method was applied to measure the area under the curve (AUC).

### Cytoplasmic calcium influx assay

Following phenotypic staining, the cells were incubated with 0.5 ml staining buffer that contained 0.02 % Pluronic® F-127 and 1 μM Fura-red AM or Rhod-2 AM for 30 min in the incubator at 37 °C. Stained cells were washed with DPBS without calcium and magnesium. Fura AM stained cells were stimulated after 30 s of flow cytometric acquisition in the presence of a final concentration of 1.4 μg streptavidin, 714 ng ionomycin, 50 μg 2-APB or 14 μg thapsigargin. Data was recorded over 4 min. Rhod-2 AM stain cells were incubated for a further 12 h, prior to acquisition. Unstimulated cells were used as a baseline when assessing the stimulation response’s AUC.

Thapsigargin is a potent inhibitor for Calcium-ATPase receptors and raises cytoplasmic calcium concentration by inhibiting the ability for the cells to pump calcium into the endoplasmic reticulum (ER) [34]. 50 ug 2-aminoethoxydiphenyl borate (2-APB) was used given its inhibition of ER and IP_3_R as previously described [35]. NK receptors (NG2DA and NKp46) were cross-linked [36] to ensure co-stimulation of human NK cells to measure calcium influx. CD19 and CD21 (complement receptor CR2) on B cells are responsible for activating the Immunoglobulin M (IgM) receptor and the three proteins together make the B cell receptor complex. This complex was then stabilised by streptavidin-mediated cross-linking to induce permanent calcium influx and enhance activation of CD19^+^ B cells [37].

### Statistical data analyses

Statistical analyses were performed using IBM SPSS Statistics version 22 software (SPSS, Chicago, USA). Significance was tested by MANOVA and reported at the P < 0.05 level between CFS/ME and healthy control groups for parameters including TRPM3 protein expression, and calcium influx in B lymphocytes and NK cells. Data are given as mean ± SEM. Flowjo was employed to analyse FCS files extracted from FACSDiva 8 software (BD Bioscience). Post Hoc test was further performed to determine specifically where the significance was between healthy controls and CFS/ME. The Levene test was used to analyse homogeneity of variance between groups.

## Results

### Subject characteristics

There was no significant difference in age between healthy controls (n = 19, 48.32 ± 2.71) and CFS/ME patients (n = 17, 51.24 ± 2.14)

### Identification of TRPM3 on Natural Killer cells and B lymphocytes

The fluorescence of fluorescein isothiocyanate conjugated (FTIC) secondary antibody was employed to quantitatively measure TRPM3 surface protein expression. Forward and side scatter dot plots were utilised to identified lymphocyte population prior to gating CD56^Bright^ NK cells (CD3^−^CD56^Bright^CD16^−^), CD56^Dim^ NK cells (CD3^−^CD56^dim^CD16^+^) and total B cells (CD3^−^CD19^+^) (Fig. 1). TRPM3^+^ NK cell subsets (Additional file 2: Figure 1) and TRPM3^+^ B cells (Additional file 3: Figure 2) were further identified for CFS/ME and healthy controls.

### Comparative assessment of TRPM3 on Natural Killer cells and B lymphocytes between healthy controls and CFS/ME patients

**Fig. 2:**
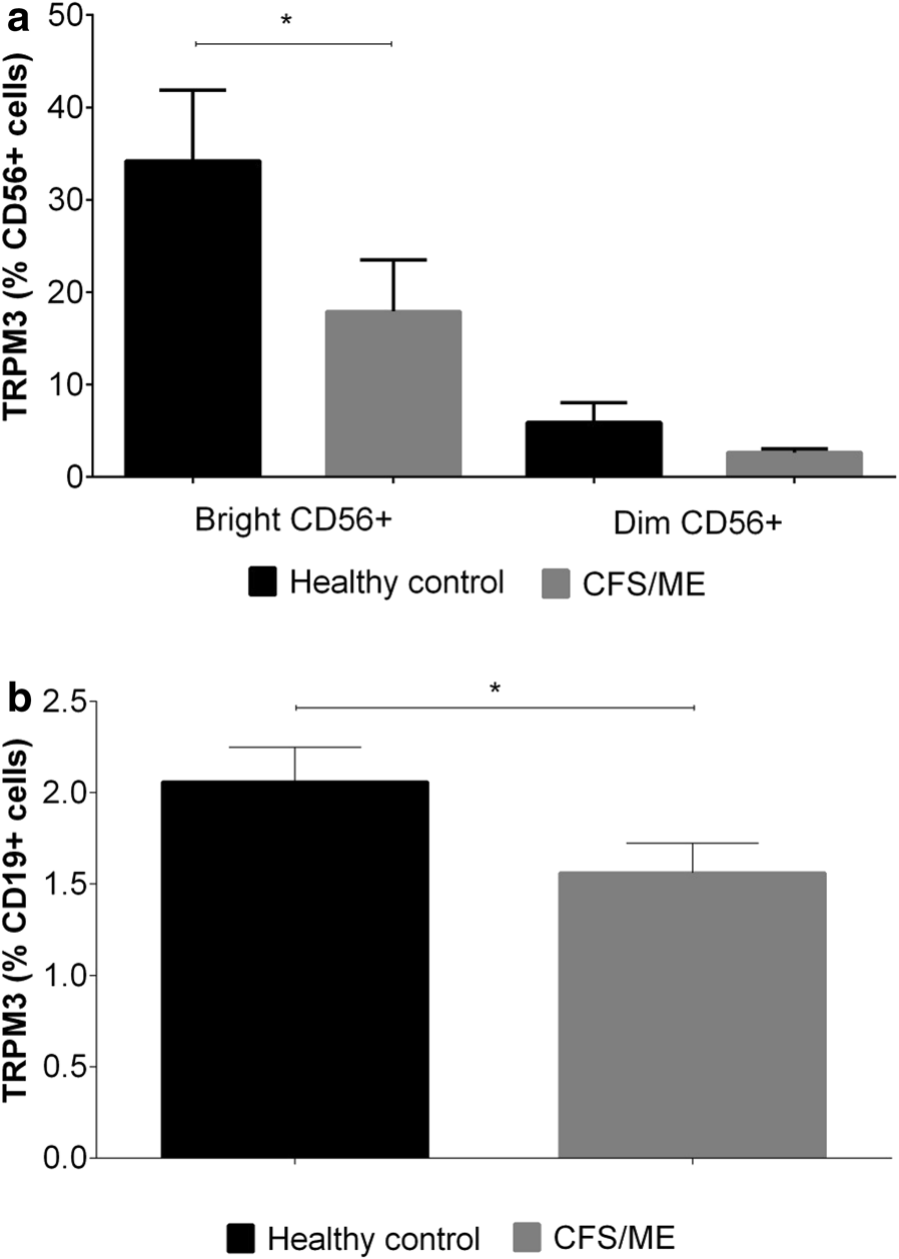
TRPM3 expression (%) on B lymphocytes and NK cells gated from HC and CFS/ME peripheral mononuclear cells. **a** NK cells subsets were charactered as CD56^Bright^ NK cells and CD56^Dim^ NK cells. Identification of TRPM3 surface expression on the NK cell subsets was analysed using indirect flow cytometry. **b** B cells were characterised as total B cells (CD3^−^CD19^+^) and indirect flow cytometry was employed to identify TRPM3 surface expression on B cells. Histograms report the means ± SEM. *Asterisk* denotes P < 0.05. *HC* healthy controls, *CFS* Chronic fatigue syndrome

CD56^Bright^ NK cells showed significantly decreased TRPM3 expression (17.37 ± 5.34 %) in CFS/ME compared with healthy controls (35.72 ± 7.38 %) (Fig. 2a). Similarly, CD19^+^ cells demonstrated significantly decreased TRPM3 expression in CFS/ME (1.56 ± SEM 0.191 %) compared with healthy controls (2.06 ± SEM 0.16 %) (Fig. 2b). CD56^Dim^ cells showed no significant difference between groups (Fig. 2a).

### Cytoplasmic and mitochondrial calcium influx

**Fig. 3:**
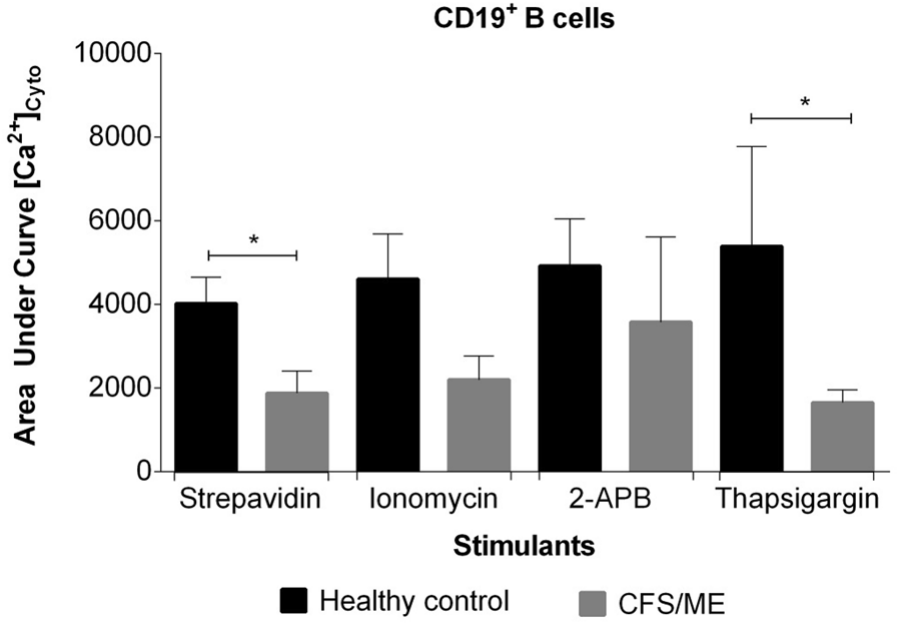
Fura-AM cytoplasmic calcium influx in CD19 + B cells. Calcium influx response curve reported as area under the curve was measure during Anti-IgM and anti-CD21 conjugated biotins were cross-linked with streptavidin or in the presence of ionomycin, 2-APB or thapsigargin. *Histograms* report the means ± SEM. *Asterisk* denotes statistically significance at P < 0.05

**Fig. 4:**
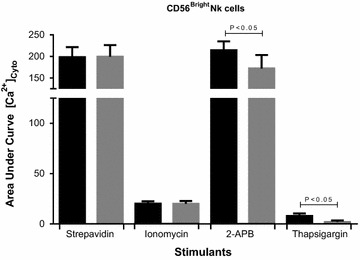
Fura-AM cytoplasmic calcium influx response during CD56^Bright^ NK cell receptors, Anti-CD314 and anti-CD335 conjugated biotins were cross-linked with streptavidin or in the presence of ionomycin, 2-APB or thapsigargin. *Histograms* report the means ± SEM. *Asterisk* denotes statistically significance at P < 0.05

Fura-2 AM and Rhod-2 AM exhibit an increase in fluorescence upon binding to cytoplasmic and mitochondrial calcium ions. Changes in mitochondrial calcium concentration in the presence of stimulants showed no significance. CFS/ME patients showed significantly reduced cytoplasmic calcium ion concentration in CD19^+^ B cells during cross-linking of CD21 and IgM receptor (P < 0.01) (Fig. 3). CD56^bright^ NK cells demonstrated a significantly decreased cytoplasmic calcium influx in the presence of 50 μg 2-APB and 14 μg thapsigargin (P < 0.05)(Fig. 4).

## Discussion

The present paper reports, for the first time, the identification of TRPM3 surface expression on NK cells and B lymphocytes in CFS/ME patients as well as healthy controls. Further, diminished expression of TRPM3 surface expression was found on B lymphocytes and NK cells in CFS/ME patients compared with healthy controls. We also report, for the first time, a significant reduction in cytoplasmic calcium ion concentration in CD19^+^ B lymphocytes during cross-linking between anti-CD21 and anti-IgM following treatment with stepadividin or thapsigargin in CFS/ME patients. CD56^bright^ NK cells also had a significant decrease in cytoplasmic calcium in the presence of 2-APB and thapsigargin in CFS/ME patients.

Investigation of TRP ion channels expression on lymphocytes has been very limited due to the difficulties of methodology as TRP channels are in relatively low abundance and there is limited availability of specific and high-affinity antibodies. Other methods of identification of TRP include molecular genetics by assessing TRP mRNA expression or isolation of protein from plasma membrane. Also, investigations of TRP ion channel functions and their roles in disease states have been reported through voltage clamping methodology or in vitro cell lines. Knock-out animal models have also been reported to study TRP ion channels (see review: [38]). TRPM3 has been reported to be expressed on a wide variety of tissues, such as kidneys, eyes, sensory neurons of the dorsal root ganglia and the β islet cells of the pancreases [2]. Only TRPM4, TRPM5, TRPM2 and TRPM7 surface expression have been reported on B cells, bone marrow cells, splenic cells, lymph node B cells and T and mast cells [39]. However, no previous investigation has reported TRPM3 on B lymphocytes or NK cells. Our novel findings suggest TRPM3 on B lymphocytes and NK cells may also be involved in calcium-mediated roles, such as those seen with other TRP family members. These calcium-mediated TRP functions contribute to a number of cellular processes and cellular functions. These processes include regulating enzymatic function and transcription factors, lymphocyte proliferation and differentiation of naïve cells into effector and memory cells as well as the production of cytokines and chemokines (see review [8]). TRPM3 alone or in conjunction with TRPC members, members of the TRPV1 family, together with muscarinic M3 receptors [40] and calcium release activated calcium (CRAC) channels [41] may need to be considered for calcium intracellular-mediated roles.

Our preliminary research findings report a significant reduction in TRPM3 surface expression for B lymphocytes and CD56^Bright^ cells from CFS/ME patients compared with healthy controls. Our group has recently reported significant associations of SNPs, predominately in *TRPM3* genes, from NK and B lymphocytes in CFS/ME patients compared with healthy controls [10, 11, 32]. The significant reduction in TRPM3 surface protein expression in conjunction with a significant reduction in Ca^2+^ influx from CD56^bright^ NK cells suggests important functional implications. When Ca^2+^ influx is prevented or reduced an immunological synapse is not formed [42, 43]. A rise or pulsatile influx in intracellular Ca^2+^ is required in cells, such as Natural Killer cells and cytotoxic lymphocytes to initiate cell function, such as the cell lysis of a target infected cell, as well as formation of mitotic spindles for cytoskeleton movement to facilitate the secretory vesicles to fuse with the plasma membrane to ultimately form the immune synapse. Moreover, the production of lytic granules for targeting and killing infected cells is also dependent on Ca^2+^ [44].

Treatment with streptavidin cross-linked with NKD2A and NKp46, which are Ca^2+^ dependent, showed no significant reduction for cytoplasmic calcium in CD56^Bright^ NK cells. In contrast, treatment with thapsigargin or 2-APB, suggests impairment of Ca^2+^ influx and storage. Treatment with 2-APB, which is both non-selective for TRPM3 [45] and an IP_3_R antagonist [35] showed a significant difference in intracellular Ca^2+^. Treatment with thapsigargin showed a significant reduction in Ca^2+^ in the CFS/ME group compared with the healthy control group, suggesting Ca^2+^ stores may be a consequence of reduced Ca^2+^ influx into the cell, ultimately lowering the function and intracellular signalling capability of the cell. Recent research has identified that CD56^bright^ NK cells are very likely precursor cells of the CD56^dim^ subset. CD56^dim^ NK cells have been shown to have shorter telomeres than CD56^bright^ NK cells from peripheral blood, suggesting CD56^bright^ cells are not as mature as CD56^dim^ [46]. Additionally, purified CD56^bright^ CD16^−^ NK cells have been shown to differentiate into CD56^dim^ cells that have the characteristic phenotypic and functional features of peripheral blood CD56^dim^ NK cells [47]. CD56^bright^ NK cells constitutively express the high- and intermediate-affinity IL-2 receptors and expand in vitro and in vivo in response to low (picomolar) doses of IL-2 [48, 49] and after activation with IL-2 or IL-12, CD56^bright^ cells exhibit similar or enhanced cytotoxicity against NK targets compared with CD56^dim^ cells [50]. We and others have shown significant reductions in CD56^bright^ NK cells in CFS/ME patients, significant reductions in lytic function of CD56^bright^ NK cells as well as significantly reduced lytic granules [16, 18, 26, 51] in CFS/ME patients.

Importantly, cross linking anti-IgM and anti-CD21 initiates a calcium-dependent pathway through transmembrane proteins, most notably Igα (CD79a) and Igβ (CD79b). B cell linker protein (BLNK) binds to tyrosine of Igα via the intracellular mechanism of crosslinking Igα and Igβ cytoplasmic tails and phosphorylation of the immunoreceptor tyrosine-based activation motif (ITAM) tyrosines which occurs by Src-family tyrosine kinases (SFTKs) and/or Syk, ultimately leading to calcium influx and protein kinase C (PKC) activation [52]. In the present study, B lymphocytes treated with streptavidin showed a significant reduction of intracellular Ca^2+^. In contrast, treatment with 2-APB showed no significant reduction in intracellular Ca^2+.^ However, treatment with thapsigargin showed a significant difference in intracellular Ca^2+^. One rationale for this outcome may be due to calcium signalling by other TRP families identified, for example TRPM4, TRPM5 and TRPM2 that have been identified on B cells, bone marrow cells and lymph node B cells. It is feasible these other TRP subfamilies [39, 53], may play a role in intracellular Ca^2+^ influx and storage. Interestingly, muscarinic acetylcholine receptors have been found to inhibit TRPM3 via the action of phospholipase C [40]. Such interaction may provide additional complexity to Ca^2+^ cell signalling as we have recently reported a significant association of SNPs in genes of muscarinic acetylcholine receptors M3 from the same cohort of CFS/ME patients who had significant association of SNPs in their *TRPM3* genes from peripheral blood mononuclear cells [54]. Future investigations comparing cell subsets that do not express TRPM3 need to be explored. Additionally, pathways that potentially influence calcium influx may discern whether reduced TRPM3 has a role in reduced intracellular calcium ion concentration.

## Conclusions

Our novel discovery of TRPM3 on NK and B lymphocytes and their significantly reduced expression in CFS/ME patients, together with the preliminary discovery of impaired Ca^2+^ cytoplasmic concentration in these cells, suggests impaired Ca^2+^ metabolism may be implicated as a novel pathway for pathogenesis of CFS/ME. Further investigation may elucidate intracellular Ca^2+^ signalling pathways and the potential role this may play on transcriptional factors, such as NFκB and NFAT as both are activated by calcium through the calmodulin dependent protein kinases and phosphatases. Moreover, as TRPs are widely expressed on almost all human cells and tissues, this raises the question as to whether similar changes of TRPM3 expression or function of these receptors promotes more widespread disruption of intracellular signalling homeostasis in CFS/ME patients.

## Additional files

10.1186/s40659-016-0087-2 The cell populations CD56 ^Bright^, CD56 ^Dim^ and CD19^+^ cells expressing TRPM3 as percentage of parent cells (%) and Mean Fluorescence Intensity (MFI).
10.1186/s40659-016-0087-2 Dot plots gating strategies for TRPM3 expressed on NK cells. Identification of NK cell subsets and TRPM3 positive in healthy controls (a.) and CFS/ME patient (b.) that are based on the isotype controls (c.).
10.1186/s40659-016-0087-2 Dot plots gating strategies for TRPM3 expressed on B cells. Identification of B cell subsets and TRPM3 positive in healthy controls (a.) and CFS/ME patient (b.) that are based on the isotype controls (c.).

## Abbreviations

2-APB: 2-aminoethoxydiphenyl borate
AUC: area under the curve
Ca2+: calcium
CFS/ME: Chronic fatigue syndrome/Myalgic encephalomyelitis
CNS: central nervous system
CRAC: calcium release activated calcium
EDTA: ethylenediaminetetraacetic acid
ER: endoplasmic reticulum
FTIC: fluorescein isothiocyanate conjugated
ITAM: immunoreceptor tyrosine-based activation motif
MANOVA: multivariate analysis of variance
NK: Natural Killer cell
PBMCs: peripheral blood mononuclear cells
PBS: phosphate buffer saline
SFTK: src-family tyrosine kinases
SNP: single nucleotide polymorphisms
TRPA: Transient receptor potential ankyrin
TRPC: Transient receptor potential canonical
TRPM: Transient receptor potential melastatin
TRPM3: Transient receptor potential melastatin subfamily 3
TRPML: Transient receptor potential mucolipin
TRPP: Transient receptor potential polycystin
TRPV: Transient receptor potential vanilloid
