# Supplementary material for: Novel identification and characterisation of Transient receptor potential melastatin 3 ion channels on Natural Killer cells and B lymphocytes: effects on cell signalling in Chronic fatigue syndrome/Myalgic encephalomyelitis patients
Source: Biol Res. 2016 May 31;49:27. doi: 10.1186/s40659-016-0087-2 (PMC4888729; doi:10.1186/s40659-016-0087-2)
Supplement: Supplementary file 1 — 10.1186/s40659-016-0087-2 The cell populations CD56 Bright, CD56 Dim and CD19+ cells expressing TRPM3 as percentage of parent cells (%) and Mean Fluorescence Intensity (MFI). [file 40659_2016_87_MOESM1_ESM.docx]

**Supplementary Table 1**

**Table S1:**

The cell populations CD56 ^Bright^, CD56 ^Dim^ and CD19^+^ cells expressing TRPM3 as percentage of parent cells (%) and Mean Fluorescence Intensity (MFI).

|  | Healthy Controls | | | CFS/ME patients | | |
| --- | --- | --- | --- | --- | --- | --- |
|  | CD56 ^Bright^ | CD56 ^Dim^ | CD19^+^ | CD56 ^Bright^ | CD56 ^Dim^ | CD19^+^ |
| TRPM3+ % | 0.1% | 14.3% | 93.0% | 83.3% | 3.0% | 96.7% |
| MFI TRPM3+ | 4654 | 9056 | 4, 314 | 2624 | 3902 | 4, 314 |
| TRPM3- % | 99.0% | 35.7% | 7.0% | 16.7% | 97.0% | 3.3% |
| MFI TRPM3- | 834 | 1708 | 35, 189 | 1037 | 737 | 34, 024 |
