# Supplementary figures and images for: Novel identification and characterisation of Transient receptor potential melastatin 3 ion channels on Natural Killer cells and B lymphocytes: effects on cell signalling in Chronic fatigue syndrome/Myalgic encephalomyelitis patients
Source: Biol Res. 2016 May 31;49:27. doi: 10.1186/s40659-016-0087-2 (PMC4888729; doi:10.1186/s40659-016-0087-2)

**Supplementary Figure S1**


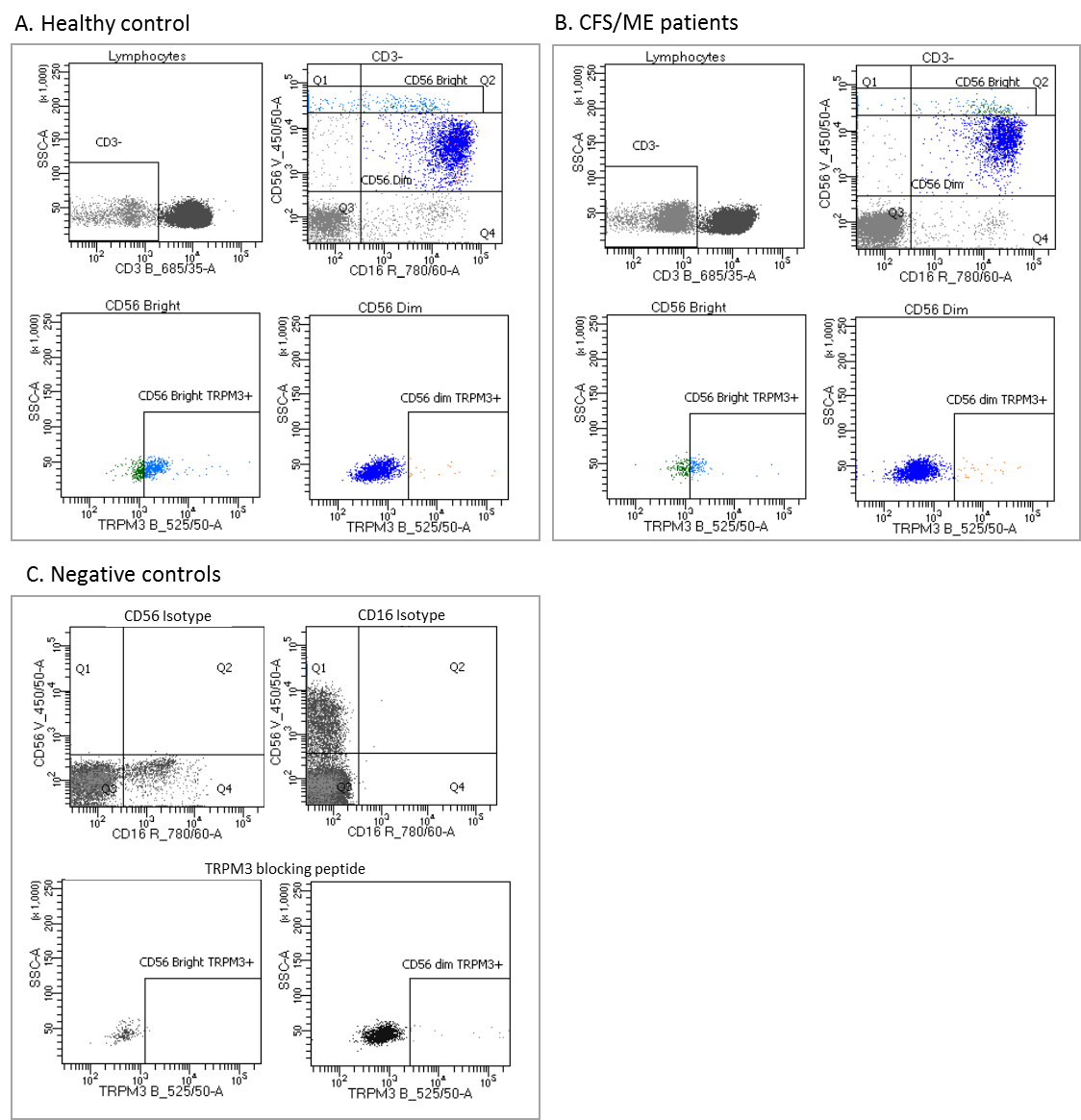

Supplement: Supplementary file 2 — 10.1186/s40659-016-0087-2 Dot plots gating strategies for TRPM3 expressed on NK cells. Identification of NK cell subsets and TRPM3 positive in healthy controls (a.) and CFS/ME patient (b.) that are based on the isotype controls (c.). [file 40659_2016_87_MOESM2_ESM.docx]

**Supplementary Figure S2**


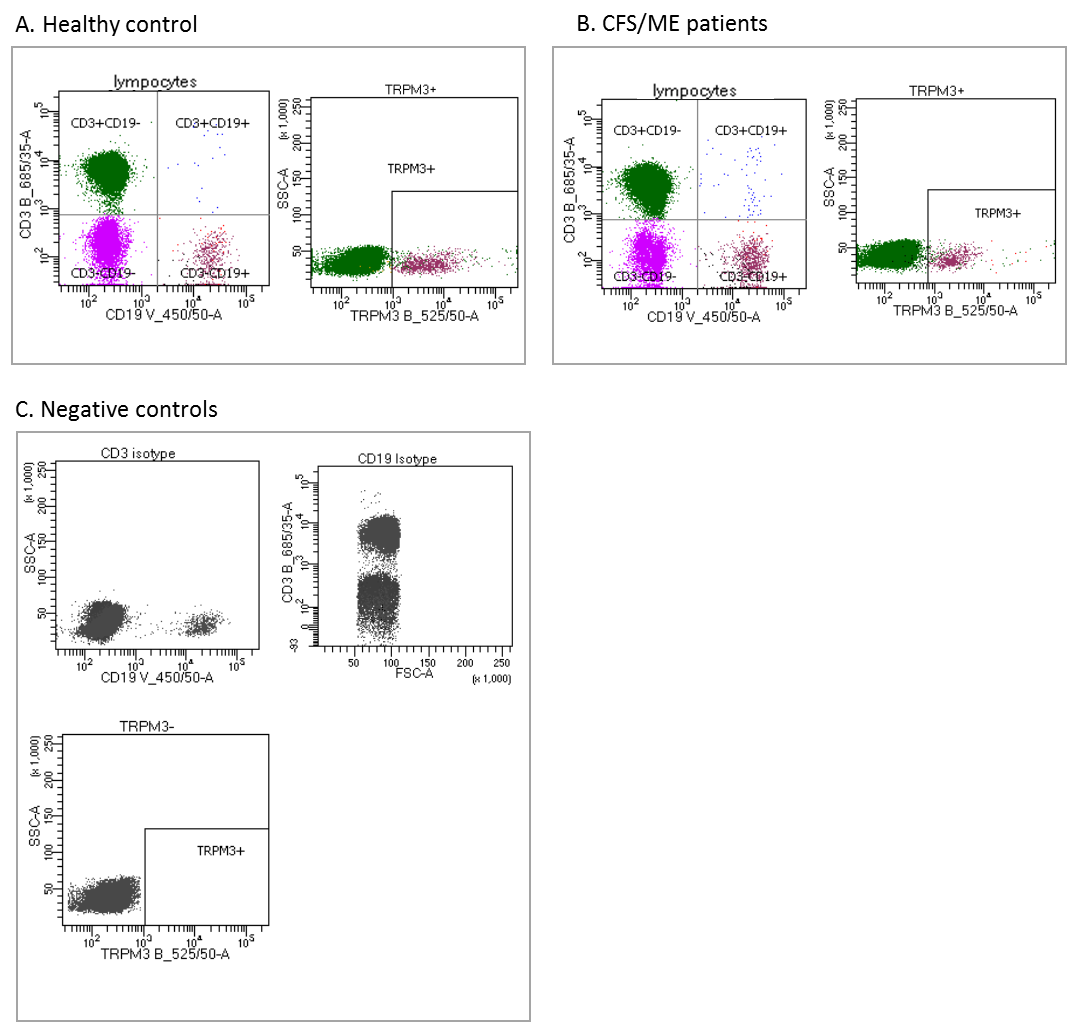

Supplement: Supplementary file 3 — 10.1186/s40659-016-0087-2 Dot plots gating strategies for TRPM3 expressed on B cells. Identification of B cell subsets and TRPM3 positive in healthy controls (a.) and CFS/ME patient (b.) that are based on the isotype controls (c.). [file 40659_2016_87_MOESM3_ESM.docx]
